# Modular Bioreactor Design for Directed Tendon/Ligament Tissue Engineering

**DOI:** 10.3390/bioengineering9030127

**Published:** 2022-03-21

**Authors:** Axel J. Delakowski, Jared D. Posselt, Christopher T. Wagner

**Affiliations:** Department of Biomedical Engineering, The College of New Jersey, Ewing, NJ 08628, USA; delakoa1@tcnj.edu (A.J.D.); jposselt1@gmail.com (J.D.P.)

**Keywords:** tendon, ligament, mesenchymal cells, differentiation, extracellular matrix, bioreactor

## Abstract

Functional tissue-engineered tendons and ligaments remain to be prepared in a reproducible and scalable manner. This study evaluates an acellular 3D extracellular matrix (ECM) scaffold for tendon/ligament tissue engineering and their ability to support strain-induced gene regulation associated with the tenogenesis of cultured mesenchymal stromal cells. Preliminary data demonstrate unique gene regulation patterns compared to other scaffold forms, in particular in Wnt signaling. However, the need for a robust bioreactor system that minimizes process variation was also evident. A design control process was used to design and verify the functionality of a novel bioreactor. The system accommodates 3D scaffolds with clinically-relevant sizes, is capable of long-term culture with customizable mechanical strain regimens, incorporates in-line load measurement for continuous monitoring and feedback control, and allows a variety of scaffold configurations through a unique modular grip system. All critical functional specifications were met, including verification of physiological strain levels from 1–10%, frequency levels from 0.2–0.5 Hz, and accurate load measurement up to 50 N, which can be expanded on the basis of load cell capability. The design process serves as a model for establishing statistical functionality and reliability of investigative systems. This work sets the stage for detailed analyses of ECM scaffolds to identify critical differentiation signaling responses and essential matrix composition and cell–matrix interactions.

## 1. Introduction

Tendon and ligament injuries represent a significant component of musculoskeletal injuries [1,2]. However, these tissues tend to heal poorly, likely because of the mechanical nature of the tissues. Although conservative treatment for musculoskeletal soft tissue injuries is commonly the first treatment approach, acute rupture typically requires surgical correction. Primary repairs are most common in tendons and can be successful depending upon location, injury severity, time to surgery, and rehabilitation protocol [2]. Similarly, many ligament injuries can be treated conservatively or with primary surgical repair, but differential healing rates and unique biological healing responses limit timely and complete restoration of joint stability [3]. In the extreme case of the anterior cruciate ligament (ACL), conservative non-operative treatment and primary repair with sutures both fail to provide clinically acceptable results in active individuals but may be acceptable in a subset of patients [4,5,6,7,8]. Therefore, reconstruction using autografts or cadaveric allografts is the primary treatment, making the ACL the most surgically corrected ligament [9,10,11]. However, current graft options all have clinical concerns, including donor site morbidity, soft-tissue endpoint attachment, integration in the osseous tunnel, possible disease transmission, reduced mechanical characteristics, and slow graft incorporation and remodeling [12,13,14,15,16].

These complications continue to drive attempts to develop functional tissue-engineered grafts for tendon and ligament reconstruction applications. Since Leung et al. first demonstrated that mechanical strain stimulates extracellular matrix (ECM) production in smooth muscle cells [17], numerous studies have demonstrated that cells sense and respond to mechanical stimuli, and that tissue structure-function relationships are heavily influenced by the mechanical environment [18]. Consequently, bioreactors that support a proper growth environment and mechanical cues have been instrumental in advancing tissue engineering (TE). Over the last few decades, bioreactor development for tendon and ligament TE has advanced from 2D systems [19] to 3D stretch and perfusion systems [20] driven by a need for more comprehensive tools to support basic biological research and translational TE construct development [21,22]. Many bioreactor systems are designed for research with specific scaffold configurations and conditions and are not validated for functional performance. While such systems can advance biological understanding, they lack versatility, and their focus on research limits translation for clinical use. Prior studies using 2D monolayers and 3D hydrogel/denatured collagen scaffolds have shown that mesenchymal stromal cells (MSCs, also commonly referred to as mesenchymal stem cells) differentiate when subjected to mechanical strain, as indicated by early genetic biomarkers [23,24], but achieving adequate mechanical performance remains elusive [20], possibly because of limitations in scaffold composition and structure. Acellular tissue-derived scaffolds retaining native ECM composition and 3D architecture (referred to herein as native 3D ECM scaffolds) [25] are extensively used clinically as biological meshes for tissue replacement and support [26,27,28,29]. Despite having randomly oriented collagen fibers, scaffold integration and ECM alignment have been observed in numerous studies [30,31], including ligament reconstruction studies [32]. However, such scaffolds exhibit specific challenges. Cell infiltration can be limiting [33], and 3D strain profiles are dependent on methods whereby mechanical forces are applied, making causal relationships between biological responses and mechanical stimuli difficult [34].

The ideal TE approach is to drive biological responses as close as possible to natural development processes using scaffold materials that are optimally engineered for the target tissue. The premise of the current study is that native 3D ECM scaffolds that have shown success in clinical settings can be used to understand normal development processes. The work herein presents initial studies using a native 3D ECM scaffold to analyze MSC responses to cyclic strain for early markers of tenogenic differentiation, testing the hypothesis that such scaffolds elicit cellular responses uniquely and differently from scaffolds that have non-native ECM architecture because of denaturation or solubilization preparation methods. While the findings support this hypothesis, they simultaneously highlight the need for a robust bioreactor system for mechanosensitive TE. Design and verification testing of a system that provides continuous monitoring and control are reported. The design process employed supports simple translation from research to development activities and can serve as a model for bioreactor design that ensures reliable and reproducible functionality.

## 2. Materials and Methods

### 2.1. MSC Strain-Induction Analysis

Non-crosslinked porcine-derived acellular dermal matrix (PADM) was prepared as previously described [25] and used as a native 3D ECM scaffold. Rat MSCs (Invitrogen, Carlsbad, CA, USA) were expanded and recovered from propagation plates in growth media (Dulbecco’s Modified Eagle Medium with 1 g/L glucose and 10% fetal bovine serum) to generate a stock suspension that was counted using standard trypan blue exclusion hemocytometric methods. An appropriate volume to achieve 10,000 cells/cm^2^ was pipetted onto collagen I-coated silastic UniFlex culture plates (Flexcell International, Burlington, NC, USA) or PADM cut to 1.5 cm by 2.3 cm, the same dimensions as the UniFlex plate culture area. To ensure uniform cell distribution, the PADM was placed in a custom, sterilizable Teflon fixture with wells having the same cross-sectional area as the PADM, and seeding media containing the appropriate cell number was pipetted on top (shown in supplemental materials). Seeded substrates were cultured in a defined growth medium for 24 h to allow cell attachment prior to starting strain regimen experiments. Following attachment, the PADM was aseptically attached to the silastic membrane of an uncoated UniFlex plate with a flexible cyanoacrylate adhesive.

5% cyclic strain at 0.2 Hz for four equally spaced 1-h periods per day was applied for 24, 48, or 72 h to bonded PADM and 2D control UniFlex plates using a Flexcell^®^ FX-5000TM system (Flexcell International, Burlington, NC, USA). Strain was applied on the basis of known strain gradient profiles within the PADM to achieve an average 5% strain level [34]. Experimental and control strain samples were compared to time-zero static controls. Following treatment, total cell RNA was isolated using Invitrogen TRIzol^®^ Reagent (ThermoFisher, Waltham, MA, USA) as described elsewhere [35], quantified by 260/280 nm UV spectrophotometry (Nanodrop, Thermofisher, Waltham, MA, USA), and analyzed by RT-qPCR. cDNA was synthesized with Invitrogen SuperScriptTM First-Strand System (ThermoFisher, Waltham, MA, USA) followed by 50 cycle amplification (Stratagene eMx3005P thermocycler, Agilent Technologies, Santa Clara, CA, USA) using Invitrogen SYBR GreenER Universal Master Mix (ThermoFisher, Waltham, MA, USA) and primers for genes of interest (Table 1). All conditions were tested in triplicate and all PCR reactions were run in triplicate for each cDNA sample and gene. Fold changes in gene expression levels, normalized to GAPDH expression, were calculated using the comparative C_T_ method [36] and reported as a percent of 2D static control levels. Data were compared to 2D strain control or static equivalent substrate condition for significant changes using either a two-sample *t*-test or ANOVA with post hoc analysis, as appropriate.

### 2.2. Bioreactor System Design

On the basis of the experimental challenges experienced during the MSC study, specific improvements in the bioreactor system were identified as essential to reliably investigate biological responses on intact ECM scaffolds. To ensure that the bioreactor met all requirements and functions reproducibly, it was designed within a design control standards framework required for US medical device development [37]. The primary design criteria are presented in Table 2.

All parts were designed in standard computer-aided design (CAD) software and checked for fit in assembly files. All media-contact bioreactor parts were manufactured from biocompatible and sterilizable materials; polyoxymethylene (Delrin^®^, DuPont, Wilmington, DE, USA) and 316 stainless steel. A key component included on each bioreactor unit is a miniature threaded in-line load cell (LCM 300, Futek, Irvine, CA, USA) that allows real-time mechanical analysis and control feedback based on scaffold changes over time. Polycarbonate sheets were used for the incubator shell to maintain environmental conditions. Custom brackets were 3D-printed from design files in acrylonitrile butadiene styrene using an Object350 FDM printer (Stratasys, Eden Prairie, MN, USA) to position supporting equipment (pump, motor, and heater).

System input and control were implemented in LabVIEW with a myDAQ data acquisition module (version 2019 SP1, National Instruments, Austin, TX, USA). The program includes a user-friendly interface to enter custom strain regimen profile parameters, including strain level, strain cycle frequency, strain period duration, rest period duration between strain periods, sample gage length, and total number of strain cycles. Additional control parameters enable environment regulation, including cell culture media exchange and setpoints for %CO_2_ and temperature levels that are maintained through PID control. In summary, the bioreactor processes are automated through the controller software so that experiments can be run for any length of time and modified at any point in time without requiring direct contact after loading seeded scaffolds until the experimental time is completed.

### 2.3. Design Verification Testing

Verification testing was conducted to confirm that the device meets the design input specifications and accurately achieves full range coverage where applicable. Specifically, this included testing for the first three inputs in Table 2. The remaining inputs inform the physical design and are addressed in the Results, below.

Strain testing was performed by programming the system to force the motor to move the tissue grips to positions corresponding to target strain values of 1%, 5%, and 10% assuming graft lengths of 10 mm, 26 mm, and 52 mm, respectively. These settings represent the minimum, average, and maximum conditions the bioreactor must be able to cover. Measurements were taken using digital calipers on three separate setups of the system (*n* = 3). These data were used to estimate the sample size required to achieve 80% power (*n* = 6, *n* = 4, and *n* = 3, respectively) and measurements resumed to complete data collection. The data were compared to target values by a one-sample *t*-test.

Similarly, frequency testing was performed by setting the motor to oscillate between 0 and 10% strain assuming a 52 mm graft length, considered to be the worst case, most challenging scenario) at frequencies of 0.2 Hz, 0.33 Hz, and 0.5 Hz. Again, the settings represent the minimum, average, and maximum conditions the bioreactor must be able to cover. Cycles were counted over 2-min intervals to determine the frequency. As above, testing was conducted on three unique setups to estimate sample size to achieve 80% power (*n* = 3, *n* = 3, and *n* = 4, respectively) and then resumed until complete. The data were compared to target values by a one-sample *t*-test.

Load cell testing was performed by running tension regimens on a mechanical testing frame and replicating the regimens through the bioreactor. Twenty-millimeter nylon grafts were tension tested at 10% strain, producing 50 N ultimate tensile strength (*n* = 4). The bioreactor motor was then programmed to apply the same regimen with load cell measurements taken in real-time. This regimen represents the maximum strain conditions and demonstrates the calibration and functionality of the load cell. The bioreactor load cell data were compared to the testing frame (Instron Model 5967, Instron, Norwood, MA, USA) load cell data by a 2-way ANOVA with replication.

One of the key design features included in the bioreactor is a “lock and key” style T-slot adaptor integrated into the reactor chamber. This feature allows any stainless-steel grip fixture to be secured in the reactor simply by sliding it onto the T (see design Results, below) with minimal manipulation that may affect the grip–scaffold interaction, scaffold integrity, or system sterility. To ensure this design was stable, ANSYS computer-aided engineering simulation software (ANSYS, Inc., Canonsburg, PA, USA) was used to apply a tensile load to the T and determine stress and deformation. The applied load, 688 N, is equal to the maximum load defined in the design inputs, and the ANSYS simulation used computer-controlled meshing and convergence parameters. Maximum stress was compared to yield stress for the chamber material to define stability.

### 2.4. Statistics 

Data are presented as means ± standard deviation. One-sample *t*-tests, two-sample *t*-tests, and ANOVA tests were conducted as indicated for specific methods and comparisons. In all cases, significant differences were determined by *p* < 0.05.

## 3. Results

**Figure 1:**
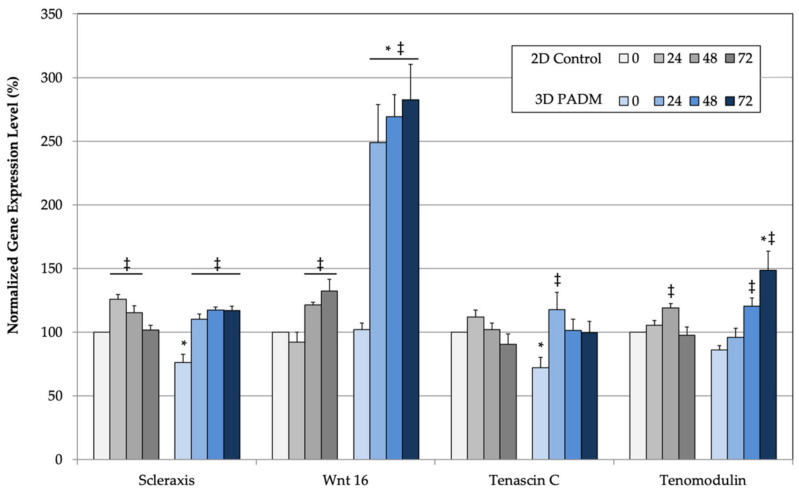
Strain-induced gene expression at 0, 24, 48, and 72 h for collagen-coated 2D membranes and 3D native ECM scaffolds using 2D static conditions as 100%. * or ‡ indicate significant (*p* < 0.05) change in expression compared to time-based 2D strain control or static equivalent substrate condition, respectively.

This work began to test the hypothesis that native 3D ECM scaffolds support differentiation differently than non-native ECM scaffolds under the premise that the responses more closely represent normal tissue development patterns. Four genes that are activated by strain and are early indicators of tenogenesis in prior studies were chosen for this preliminary study. PCR primer controls showed amplification of a single product by melt analysis (not shown). Normalized gene expression profiles showed low transient increases in the 2D cultures (Figure 1). These responses are consistent with published studies using scaffolds lacking native ECM composition and organization [23,24]. Normalized gene expression profiles in the 3D cultures were initially reduced compared to 2D culture, which is consistent with prior work showing reduced cell proliferation in 3D hydrogel culture [23]. Since expression levels were initially reduced, the percent increase was greater in 3D cultures than in 2D cultures.

All cellular biomarkers were significantly elevated and exhibited sustained gene expression levels following strain exposure when cultured on a native 3D ECM scaffold. Scleraxis and tenascin C showed early increases that were sustained throughout the study. By contrast, Wnt 16 and tenomodulin exhibited significant time-dependent increases with respect to both static and 2D configuration controls (Figure 1).

**Figure 2:**
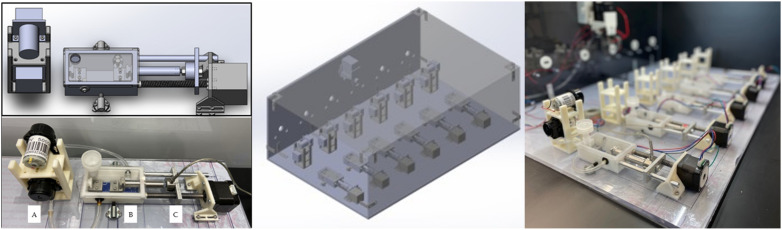
CAD assembly of single bioreactor unit (**left-top**) and a mounted bioreactor unit prototype (**left-bottom**) consisting of (**A**) pump assembly, (**B**) bioreactor assembly, and (**C**) load cell assembly. CAD assembly (**center**) and prototype under construction (**right**) of the full six-chamber reactor system.

Despite the unique and significant strain-induced mRNA responses in MSCs cultured on native 3D ECM scaffolds, challenging issues occurred with the strain model. These issues highlighted the need for a robust, versatile, reproducible, and easily controlled bioreactor. On the basis of the functional requirements presented in Table 2, a modular bioreactor system was designed and prototyped wherein each bioreactor chamber is mechanically and environmentally isolated (Figure 2). Key features include an integrated pump system to remove and replenish culture media without disrupting system integrity, a T-slot adaptor to attach grips specific to scaffold configuration allowing versatility in TE construct design, an in-line load cell to measure mechanical performance throughout the culture process and control the mechanical stimulation profile on the basis of mechanical changes, and, finally, a chamber-specific motor driver. Isolating each chamber and its system components allows modularity in experimental design and enables simple part change-out in case repair is needed.

**Figure 3:**
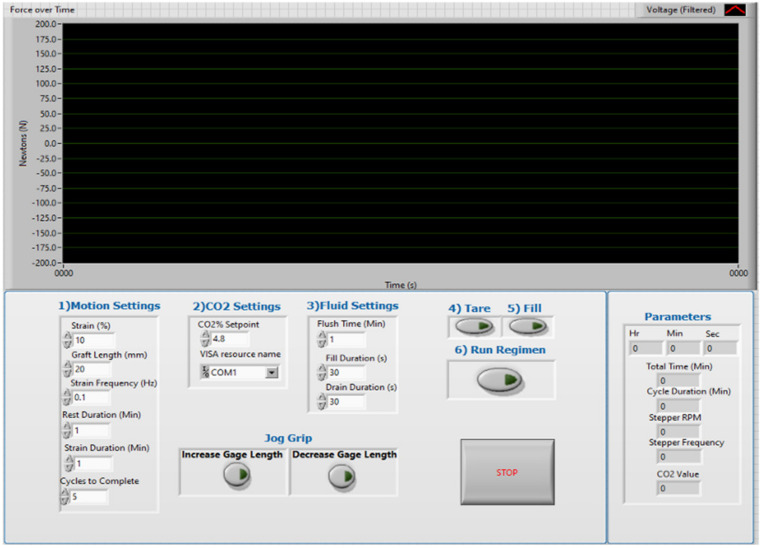
Controller graphical user interface showing inputs to define strain regimen, environmental settings, culture media replenishment fluid settings, and the mechanical load monitor.

To control the overall bioreactor system, a simple graphical user interface was programmed in conjunction with the backend control algorithm to regulate the strain regimen for each bioreactor chamber (Figure 3). This interface also shows the tensile load developed in the scaffold during each cycle of the strain period, which can be used to modify the strain profile as needed without disrupting the overall system or individual reactor chamber integrity. The specific calibration parameters for each load cell are entered into the controller algorithm ensuring accurate load measures.

**Figure 4:**
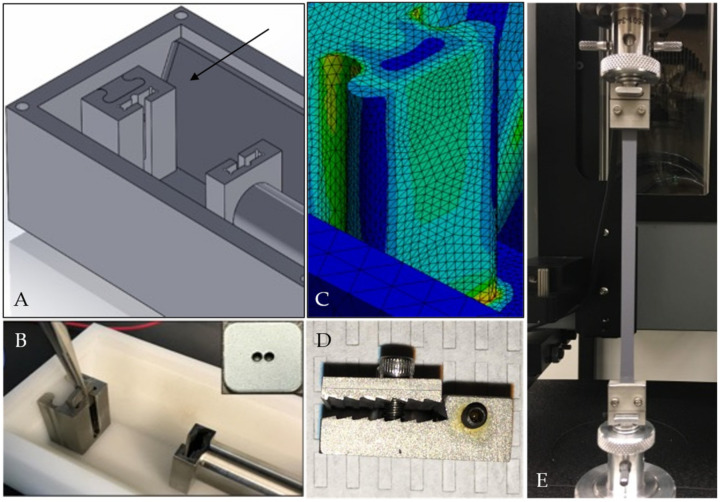
CAD assembly (**A**) and as-built image (**B**) of culture chamber showing T-slot adaptor with a stainless-steel button grip installed (arrow). The button for securing polymer scaffolds is shown in panel B inset. ANSYS analysis of T-slot adapter showing overall stress under maximum design load (**C**). Soft-tissue grips that fit the same T-slot adaptor are shown in detail (**D**), installed in the mechanical testing frame on a custom T-adaptor (**E**). See Figure 2 for the soft-tissue grips installed in the bioreactor chamber.

As noted above, one of the key features of the design is a versatile T-slot adaptor to attach scaffold-specific grips. Two distinct grip designs were constructed, a serrated system to hold soft tissue-based intact ECM scaffolds and a button system to culture polymer fiber-based scaffolds (Figure 4). This T-slot adaptor must remain stable while in use to allow accurate strain development in the attached scaffold. ANSYS structural analysis calculates a maximum equivalent stress development of 30.7 MPa within the adaptor structure, which is less than the yield stress of polyoxymethylene homopolymer at 25 °C and 37 °C, 69 MPa, and 61 MPa, respectively.

**Figure 5:**
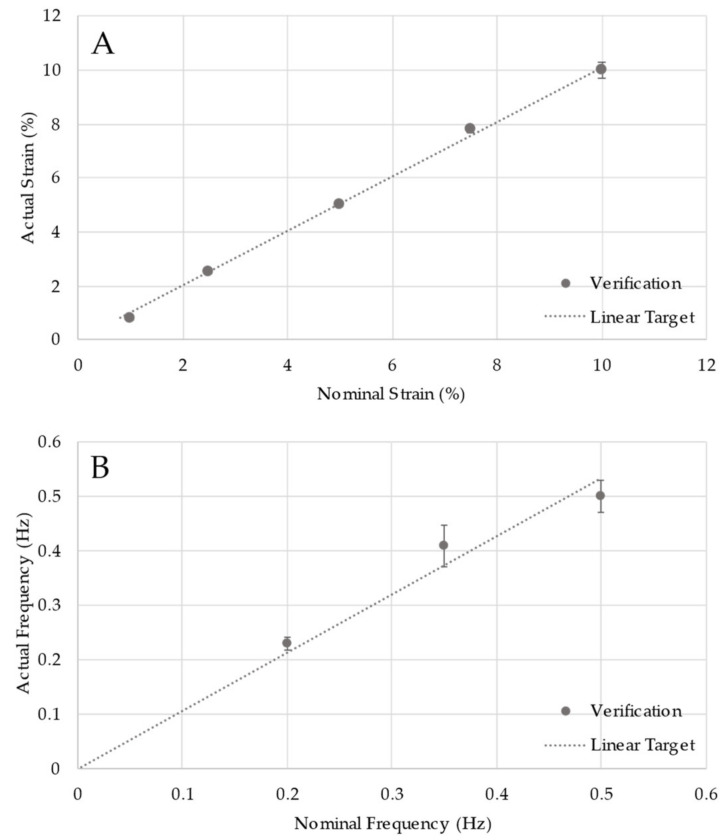
Verification for strain (**A**) and frequency (**B**). Datapoints represent multiple measures, as indicated in the methods. The linear target line represents the line of perfect concordance.

Although the system as designed meets the size and configuration design criteria, specific verification testing is needed to demonstrate that the system functions accurately across all specification ranges. This testing found no significant difference between nominal (programmed) and actual strain or cycle frequency (Figure 5). Most importantly, the line of perfect concordance lies within the 95% confidence interval of the data, indicating statistical agreement across the functional range for both strain and cycle frequency.

**Figure 6:**
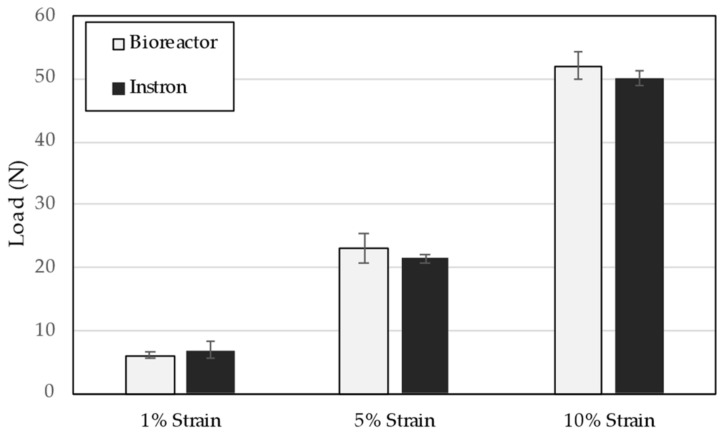
Verification of load cell measurement compared to calibrated mechanical testing frame. No significant differences (α = 0.05) between measurement modes were observed at any strain level (*n* = 4 for all conditions).

Verification testing on the in-line load cell also demonstrated no significant difference between the load cell measures and those from a calibrated mechanical testing frame using a standard polymer fiber as the test article (Figure 6).

## 4. Discussion

Treating tendon and ligament injuries represent a significant clinical burden. Conservative treatment and primary repair for most tendon and ligament injuries are successful in restoring mechanical stability, but healing tends to be slow and mechanically inferior [2,3]. ACL ruptures represent one of the most challenging ligament injuries. It is estimated that ACL tears occur in 1 in 3000 people in the United States annually [41] and that more than 100,000 occurrences require surgical reconstruction [10,42]. However, these statistics may underestimate the number and distribution of surgical reconstructions [43]. Despite the complications associated with autografts and allografts, rates of reconstruction are increasing as older adults are staying active longer [44] and children are entering highly competitive sports earlier [45,46]. Overall, the need for grafts to treat tendon and ligament injuries with fewer complications and that do not create secondary deficits continues to drive research and development of off-the-shelf reconstruction alternatives.

Working from the premise that a native 3D ECM scaffold would induce biological responses more closely resembling natural development, the results presented here demonstrate that such scaffolds support mechanotransduction to MSCs yielding unique gene expression profiles as compared to 2D cultures and 3D hydrogel cultures, both of which have non-native organization. The signaling molecules analyzed in this preliminary study have been shown to be upregulated in MSCs by mechanical forces. In particular, scleraxis and tenomodulin are expressed in tendon and ligament tissues and upregulated as late markers of tenogenesis [47,48]. Similarly, the Wnt 16 message level was upregulated in human MSCs exposed to mechanical strain [24]. However, the large response variation across MSC samples prevented the increased levels from being significantly elevated over baseline. On the basis of those reports, the observed increase in Wnt 16 signaling is not surprising, although the degree is striking. Further, the expression patterns have not been observed in 2D systems and are different than those from cells cultured in 3D collagen hydrogels having a non-native ECM architecture [23,24]. These results implicate a unique role for 3D scaffolds with specific composition and organization. The 3D native ECM scaffolds evaluated in this study have not been used for such analyses previously, so it is reasonable to expect expression differences. Whether those differences are specifically a function of ECM complexity requires additional investigation since the 2D control used in the study cannot distinguish between composition and organization.

The roles of Wnt 16 in cartilage homeostasis and damage regulation, as well as cortical bone regulation, are more established than a potential role in tenogenesis [49,50,51,52]. Wnt 16 is upregulated following cartilage injury to protect the tissue from subsequent breakdown and inhibits excessive canonical pathway activation known to cause cartilage damage [53]. More generally, Wnt signaling regulates MSC differentiation based on scaffold dimension [54], but canonical and non-canonical Wnt pathways exhibit opposing biological functions [55], suggesting a more complex signaling mechanism requiring further studies to elucidate. The involvement of other Wnt family members would not be surprising since the regulation of Wnt ligands is emerging to be highly interconnected, activating multiple pathways based on local cellular and biochemical conditions [56]. For example, the canonical Wnt/β-catenin signaling pathway regulates periodontal ligament homeostasis [57] and is correlated with graft–bone interface development [58]. While Wnt 16 signaling may be important during tendon/ligament development, a role in enthesis formation is also reasonable given known roles in cartilage regulation. It is unclear from the current work if the Wnt 16 message changes are due to tenogenesis or signaling for enthesis formation. Additional genetic and phenotypic analysis is needed to differentiate between those two possible outcomes.

This analysis suggests a possible reparative role of Wnt signaling in MSCs that is more apparent using native 3D ECM scaffolds. Thus, these scaffolds may better support strain-induced MSC differentiation to tendon/ligament in TE applications. Clinical use demonstrates that they recellularize, revascularize, and exhibit collagen transition in response to physiological cues [59,60,61,62,63,64], and preclinical studies have shown similar results as a ligament reconstruction graft [32]. Therefore, they may also help identify critical cell binding sites and organizational requirements for engineering mechanically sensitive tissues such as tendons and ligaments. The results shown here support the hypothesis that the specific composition and native 3D architecture of intact ECM is an important component of strain-induced upregulation of early markers for MSC differentiation and that additional studies examining longer culture times and a broader set of early differentiation markers are warranted.

Dermal-derived scaffolds, such as the PADM representative of native 3D ECM scaffolds used in this study, may not be unique with respect to supporting upregulation of early tenogenesis markers in MSCs. Decellularized tendon scaffolds support scleraxis up-regulation in cultured MSCs along with additional markers of tissue formation, but the increases in mechanical strength remained small and the tendon was thin-sectioned, presumably to reduce the overall thickness and enhance cellular infiltration [65]. Ultimately, in vivo performance remains to be seen given that responses to decellularized materials are highly dependent on the decellularization process [30].

Despite the unique pattern and significant levels of strain-induced gene responses in MSCs cultured on native 3D ECM scaffolds, challenging issues occurred with the strain model. First, the scaffold was bonded to the silastic membrane, through which the ECM deformation is generated. The resulting non-uniform strain patterns have been presented elsewhere [34]. Although the results presented here are based on carefully chosen strain parameters and scaffold areas for PCR analysis, this method reduces the cells sampled and the resulting genetic material collected, making subsequent PCR analysis difficult. Second, the bonding method itself imparts undesired effects. The bonding agent may impregnate the scaffold, affecting cell replication or biological responses, although cell quantification and control response did not show significant effects in the data presented. However, the large issue with this bonding mode is related to attachment permanence. Scaffolds occasionally separated from the underlying silastic membrane, which resulted in limited or no strain transmission to the scaffold. This issue forces careful checking of the bond following strain experiments and prior to cell recovery since detached samples must not be analyzed. While the data presented all came from solidly attached scaffolds, repeating experimental conditions is time-consuming and costly. Third, the intricate manipulations needed to seed scaffolds and bond them to apply mechanical stimulation can lead to sample contamination. Again, careful observation of completed stain experiments was required. Finally, the system used enables biological strain-induced responses to be analyzed. However, it does not support the formation of a viable TE construct for implantation and functional analysis. Any attempt to translate responses identified with this model to a system that can support the development of such a construct would require the detailed design and testing done here. These issues drive the need for a new system and the work presented in this report.

The approach taken with the bioreactor design described was to develop a system that would support research into the biological processes of tissue formation and the development of TE constructs that meet the tenets of functional tissue engineering [66]. The resulting engineered tissue must meet mechanical and biological benchmarks for tendon and ligament repair [67,68]. The size and capabilities support testing with scaffolds and tissues having a variety of shape factors and mechanical requirements. The versatile grip design allows for simple installation and removal without disrupting the grip–scaffold interaction. The modular grip system also allows direct transfer to function-specific mechanical frames using the unique T-adaptor design. However, the most significant value of the design control process used is the verification testing conducted to ensure robust and stable function. All critical functional specifications were met, including verification of physiological strain levels from 1–10%, frequency levels from 0.2–0.5 Hz, and accurate load measurement up to 50 N. Careful calibration of the load cell was able to avoid friction issues with measurement accuracy that is recognized to occur with sensors outside the bioreactor chamber [21].

The combinations tested during verification represent the worst-case scenarios. Since this testing demonstrated adequate performance, the system is concluded to be robust across the full range of performance metrics. This level of versatility exceeds that of previously described bioreactors, and the design combinations, modular flexibility, and robust verified functionality built into the system are unique among bioreactors systems for tendon/ligament tissues. Although some advanced systems incorporate load measuring systems, they typically are used for measuring existing tissues rather than for engineering them [69,70], lack flexible modularity, possess limited ability for long-term culture and construct maturation, and ultimately have not been able to achieve the mechanical properties for immediate function following implantation [71]. Despite these limitations, it should be recognized that not all systems were designed for full-size ligament production or scale-up and that these pioneering works established many of the scientific paradigms the current systems and research studies implement. An excellent review of bioreactor history and their use in understanding the science of mechanotransduction in tendon and ligament TE is available [22].

Finally, the verification testing conducted demonstrates that the system can robustly and reproducibly function at the setpoints. This conclusion is extremely important in that variability can inordinately affect understanding. The variation of strain gradient patterns in 3D scaffolds [34] was one of the driving factors for the new system. The potential complex and possibly opposing roles of canonical and non-canonical Wnt family members only reinforces the need for robust invariant experimental methods. Variability in graft production, whether in the materials used or bioreactor functionality, only compounds the variation inherent in animal models [40]. A high degree of system control is required to establish causal relationships between process factors and ultimate tissue function.

The focus of this report is a method for designing robust, reproducible, and reliable systems to investigate mechanical stimulation for TE applications. While the results demonstrate that the system meets the design inputs, biological validation remains to be completed. Such validation work is planned and will address some of the limitations of the MSC study reported that identified the need for the bioreactor system. Although the PADM used as a native 3D ECM scaffold is extensively characterized and used successfully in a variety of clinical applications, its exact composition is undefined. The 2D control used in the study contained only collagen I as a coating on the silastic membrane used to generate strain. This control allowed for comparison to a non-native ECM substrate, but it is not able to discriminate between the 3D structure and ECM composition as the critical component, resulting in the observed mRNA upregulation. Similarly, the PADM does not contain the aligned ECM organization found in tendon and ligament tissue. As such, it is unclear whether the signaling observed in the tenogenic markers analyzed are precursors to MSC differentiation and tendon/ligament tissue formation. More genes representative of tenogenesis, including extracellular matrix components, need to be evaluated. Interestingly, using decellularized tendon as a TE scaffold for cultured MSCs demonstrated initially reduced message levels similar to the reductions observed here on PADM, albeit for different genes of interest [65]. Despite the study limitations, the biological responses presented highlight the need for continued analysis. The biological work will be extended to long-term culture with analysis of additional tenogenic biomarkers, ECM production, and cytochemical analyses to identify cellular changes. These future studies will be a top-down approach to identify essential composition for tendon and ligament TE. The reproducible function of the bioreactor system described herein will be essential in defining causal relationships between mechanical stimulation and essential critical ECM composition.

## 5. Conclusions

Functional tissue-engineered tendons and ligaments remain to be engineered in a reproducible and scalable manner. Developing an optimized interaction of cells with a scaffold structure and mechanical stimulation to mimic developmental cues remains elusive. The data presented indicate that native 3D ECM scaffolds support upregulation of early tenogenic markers in cultured MSCs uniquely as compared to other scaffold forms. The potential that these responses may represent more normal development pathways needs to be explored further. In addition, a robust, versatile, and reproducible bioreactor system was designed and verified to continue these studies. The bioreactor accommodates 3D scaffolds with clinically relevant sizes, is capable of long-term cell culture regimens, supports continuous monitoring and feedback control, and utilizes a variety of scaffold configurations, including seeded ECM matrices and polymer fiber structures. This last facet is important in that native 3D ECM materials are challenging to engineer to ideal scaffolds because of their intrinsic structure and form factors. The design process presented serves as a model for establishing statistical functionality and reliability of investigative bioreactor systems. This work sets the stage for detailed analyses of ECM scaffolds to identify critical differentiation signaling responses and the necessary cell–matrix interactions that can then be incorporated into designed materials for truly engineered scaffold designs.

## Figures and Tables

**Table 1:** PCR primer sequences, source, and expected product size for genes analyzed.

Gene	Sense (5′ → 3′) Anti-Sense (5′ → 3′)	NCBI GenBank Accession Number	Product Size (bp)
GAPDH	CCACAGTCCATGCCATCACT TAGGAACACGGAAGGCCATG	NM_017008.4	183
Scleraxis	ACAGAAAGACGGCGATTCGA GGCCTGGGTACAAGTGTTCA	NM_001130508	249
Tenomodulin	TGCTGGATGAGAGAGGTTACTG TAGACTCTCCCAAGCATGCG	NM_022290	181
Tenascin-C	ACGGTTTCTGTCTGTCCTGG TCGTACTCAGTGGCCTCTCT	NM_053861	160
Wnt 16	CAAGAGGAAGATGCGCAGGA ACGTACGGTTGCACTCTCTG	NM_001109223	152

**Table 2:** Design inputs for modular bioreactor.

Input	Justification/Explanation
Device must apply physiological strain levels between 1–10% (accurate to 0.1%) both statically and cyclically at a frequency of 0.2–0.5 Hz (accurate to 0.01 Hz).	Physiological strain levels and those investigated for MSC differentiation cover the range specified [38]. Similarly, cycle frequencies used for cell culture systems on in the range indicated. The accuracy targets limit experimental variation.
Device must adjust to and maintain physiological temperature range of 25–42 °C (accurate within 1 °C of set point).	The specified range covers room temperature to heat shock conditions allowing for unique environmental conditions. The accuracy target limits experimental variation.
Device must measure loads up to: 200 N with 0.1 N accuracy (primary) 688 N with 0.1 N accuracy (secondary)	Specified load primary and secondary targets required to tension grafts to maximum physiological strain levels based on small animal (primary) and human (secondary) ACL. Adapted from [39] for activities of daily living. Target load defines load cell specification.
Device must allow for graft placement with minimal user manipulation and without disrupting construct-grip connection.	This requirement ensures that grip-scaffold manipulations are performed in a controlled biological safety cabinet, minimizing potential contamination. It also supports modularity to allow transfer to a mechanical testing frame for subsequent testing without disrupting the grip-scaffold connection.
Device must be sized to accommodate scaffold dimensions up to 52 mm in length and 11 mm in diameter.	Specifications are based on insertion requirements for large animal reconstruction models [40].
All tissue culture-contacting surfaces must be biocompatible and sterilizable.	This requirement is necessary for long-term cell culture.
Strain regimen and culture environmental condition setpoints must be user-specified inputs.	Specifications include environmental temperature and CO_2_ setpoints, strain cycle parameters, and cycle and culture duration parameters.

## Data Availability

Data and design schematics are available upon request to the corresponding author.
